# Versorgung von diametaphysären Unterarmfrakturen im Kindes- und Jugendalter: Die antegrade intramedulläre Nagelosteosynthese und ihre Alternativen

**DOI:** 10.1007/s00064-024-00877-3

**Published:** 2024-11-14

**Authors:** H. Rüther, C. Spering, L. Fortini, K. Dresing, W. Lehmann, T. Radebold

**Affiliations:** 1 Klinik für Unfallchirurgie, Orthopädie und Plastische Chirurgie, Universitätsmedizin Göttingen, Robert-Koch-Str. 40, 37075 Göttingen, Deutschland; 2 Fachzentrum Unfall- und Handchirurgie, Orthopädische Klinik Hess. Lichtenau, Hessisch Lichtenau, Deutschland

**Keywords:** Unterarmfraktur, Radiusfraktur, TEN – ESIN, Kind, Diametaphysär, Antegrad, Forearm fractures, Radius fractures, TEN—ESIN, Children, Diametaphyseal, Antegrade

## Abstract

**Operationsziel:**

Die Osteosynthese bei dislozierten diametaphysären Unterarmfrakturen dient der Wiederherstellung von Anatomie und Funktion. Durch die Versorgung mit einer antegraden intramedullären Nagelosteosynthese im Radius sollen Länge, Rotation und Achse im Rahmen der altersspezifischen Korrekturgrenzen wiederhergestellt werden. Die ausreichende Stabilität gewährleistet eine frühfunktionelle Nachbehandlung ohne Last.

**Indikationen:**

Dislozierte diametaphysäre Unterarm- oder Radiusfrakturen, die sich nicht geschlossen, stabil reponieren lassen oder außerhalb der altersspezifischen Korrekturgrenzen verbleiben.

**Kontraindikationen:**

Radius- oder Unterarmfrakturen, die sich distal oder proximal des definierten Areals befinden. Im Zugangsweg befindliche Weichteildefekte, Kontaminationen oder Infekte.

**Operationstechnik:**

Im Verlauf des Thompson-Zugangs wird der Soft-Spot zwischen M. extensor digitorum und M. extensor carpi radialis brevis aufgesucht und eine ca. 3–4 cm Hautinzision durchgeführt. Dann stumpfes Präparieren bis auf den Knochen unter Schonung des N. radialis profundus und superficialis. Retraktion der Muskulatur mit 2 Langenbeck-Haken. Eröffnen der Kortikalis mit einem Pfriem. Gegebenenfalls kann zuvor ein 2,5-mm-Bohrer mit Gewebeschutz bei sehr harter Kortikalis verwendet werden. Der TEN-Durchmesser (TEN = Titanium Elastic Nail) wird so gewählt, dass er etwa zwei Drittel des Markraumes ausfüllt. Es empfiehlt sich, ein Abflachen der TEN-Kufe mit einer Parallelflachzange durchzuführen. Nach geschlossener Reposition wird der TEN dann bis vor die Wachstumsfuge unter leicht rotierenden Bewegungen gebracht. Der TEN wird am proximalen Ende umgebogen und oberhalb der Muskelbäuche abgekniffen. Alternative Verfahren sind die Kirschner-Draht-Osteosynthese oder der retrograde TEN von radial oder dorsal, mit oder ohne additive Biegung.

**Weiterbehandlung:**

Ziel der Osteosynthese ist die frühfunktionelle Nachbehandlung ohne Last. Sportkarenz wird für 8 Wochen empfohlen. Die Metallentfernung kann nach Konsolidierung zwischen 3 und 6 Monaten erfolgen.

**Ergebnisse:**

Deutlich dislozierte bzw. außerhalb der Korrekturgrenzen liegende Radius- und Unterarmfrakturen im Kindesalter zeigen nach beschriebener Osteosynthesetechnik sehr gute Behandlungsergebnisse bei geringem Risikoprofil. Eine Pseudarthrose konnte genauso wie Nervenschäden nicht beobachtet werden. Eine sekundäre Dislokation trat nicht ein.

## Abstract (en)

**Objective:**

Osteosynthesis in dislocated diametaphyseal forearm fractures is intended to restore anatomy and function. Antegrade intramedullary nailing in the radius is used to restore length, rotation, and axis within the age-specific correction limits. Sufficient stability ensures early functional postoperative treatment without load.

**Indications:**

Dislocated diametaphyseal forearm or radius fractures that cannot be closed, stably reduced, or remain outside the age-specific correction limits.

**Contraindications:**

Radius or forearm fractures located distal or proximal to the defined area. Soft tissue defects, contamination or infections located in the access path.

**Surgical technique:**

In the course of the Thompson approach, the soft spot between the extensor digitorum and extensor carpi radialis brevis muscles is located and an approx. 3–4 cm skin incision is made. Then blunt preparation down to the bone, sparing the profundus and superficial radial nerve. Retraction of the musculature with two Langenbeck hooks. Opening of the cortex with an awl. If necessary, a 2.5 mm drill with tissue protection can be used beforehand if the cortex is very hard. A titanium elastic nail (TEN) diameter is selected so that it fills approximately 2/3 of the medullary canal. It is recommended to flatten the TEN runner with parallel flattening forceps. After closed reduction, the TEN is then brought up in front of the growth plate with slightly rotating movements. The TEN is bent over at the proximal end and pinched off above the muscle bellies. Alternative procedures include Kirschner wire osteosynthesis or retrograde TEN from radial or dorsal, with or without bending.

**Postoperative management:**

The aim of osteosynthesis is early functional follow-up without load. Sports abstinence is recommended for 8 weeks. Metal removal can be performed after consolidation between 3 and 6 months.

**Results:**

Clearly dislocated or outside the correction limits infantile radius and forearm fractures show very good treatment results with a low risk profile after the described osteosynthesis technique. Pseudarthrosis and nerve damage were not observed. Secondary dislocation has not occurred.

## Vorbemerkungen

**Abb. 1:**
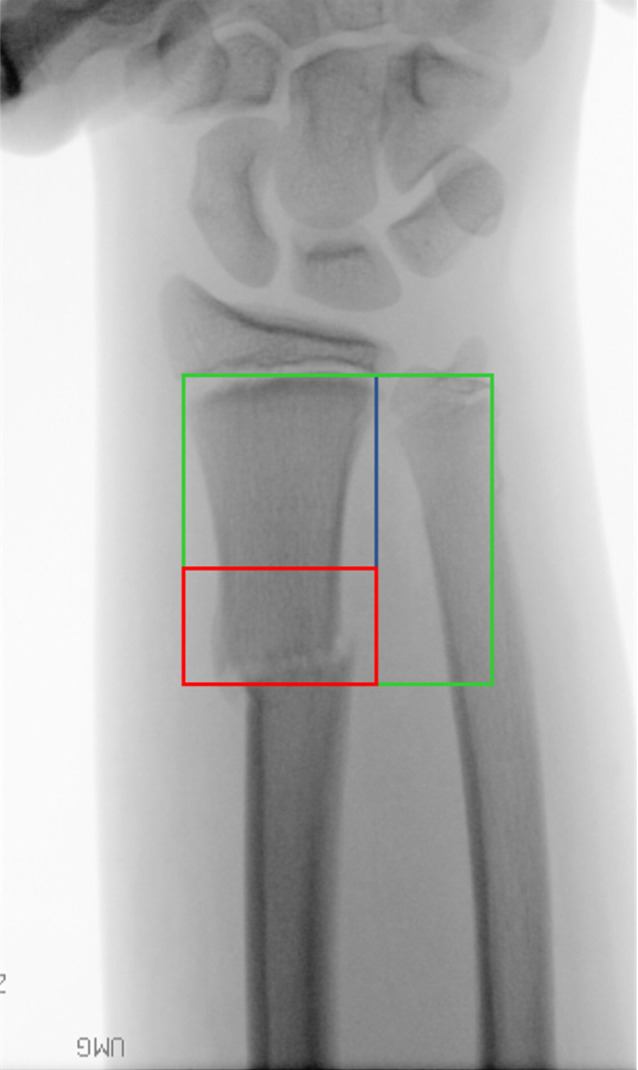
Definition der Diametaphyse (*rot*); Subtraktion des Quadrats über der Wachstumsfuge des Radius (*blau*) vom Quadrat über der Wachstumsfuge von Radius und Ulna (*grün*)

Jedes zweite Kind erleidet während des Wachstums einen Knochenbruch [1], davon betreffen ca. 54 % den Unterarm; 18 % liegen im Schaftbereich. Der überwiegende Anteil mit 77 % ist am distalen Unterarm zu finden [2]. Sind die Dislokationen so ausgeprägt, dass sie außerhalb der Korrekturgrenzen liegen bzw. lassen sich die Frakturen geschlossen nicht adäquat reponieren, ist die Operation indiziert. Während im Schaftbereich die ESIN(elastisch stabile intramedulläre Nagel)-Osteosynthese die klassische operative Versorgung darstellt [3], wird der distale Unterarm in der Regel mit Kirschner-Drähten versorgt [4]. Frakturen im Übergang zwischen Dia- und Metaphyse stellen hierbei eine Eigenart dar. Subtrahiert man vom Quadrat über beiden Wachstumsfugen am distalen Unterarm das Quadrat über der Fuge des distalen Radius alleine, erhält man den genannten Bereich (Abb. 1; [5]). Diese Region ist in der AO Pediatric Comprehensive Classification of Long-Bone Fractures (PCCF) im proximalen Anteil der Metaphyse gelegen und entspricht damit dem Code 23-M/X.X [6].

Die Standardversorgung in dieser Region stellt bisher die Kirschner-Draht-Osteosynthese oder die klassische retrograde ESIN-Osteosynthese dar.

Die Versorgung mit Kirschner-Drähten erfolgt retrograd vom Processus styloideus radii über die Wachstumsfuge in den proximalen Anteil der Fraktur. Die Stärke der Kirschner-Drähte wird in Anbetracht des Alters des Kindes zwischen 1,4 und 1,6 mm gewählt. Ein Kreuzen der Drähte auf Frakturniveau ebenso wie ein mehrfaches Bohren durch die Wachstumsfuge sollten möglichst vermieden werden. Ebenfalls muss dringend die Ranvier-Zone geschont werden, um eine Wachstumsstörung zu vermeiden. Ob 1 Draht oder 2 Drähte ausreichend sind, wird weiterhin vielerorts diskutiert. Häufig ist jedoch bei diametaphysären Frakturen eine Fixierung nicht oder nur ungenügend stabil möglich, da die Drähte in einem zu flachen Winkel eingebracht werden müssen und so nur wenig oder insuffizient die Gegenkortikalis fassen [4].

Die retrograde ESIN-Osteosynthese in klassischer Art von radial distal stellt den Standard für die Versorgung von diaphysären Radiusfrakturen dar. Hierbei kommt es zu einer 3‑Punkt-Abstützung, die eine Stabilisierung der Fraktur als Folge hat [7]. Da bei diametaphysären Frakturen häufig die Gegenkortikalis vor Frakturniveau nicht erreicht werden kann, kommt es zu einem Ausbleiben der Verspannung. Dies kann bei so verbleibender Instabilität zu einer Ad-latus-Dislokation sowie Angulation der Fraktur führen.

**Abb. 2:**
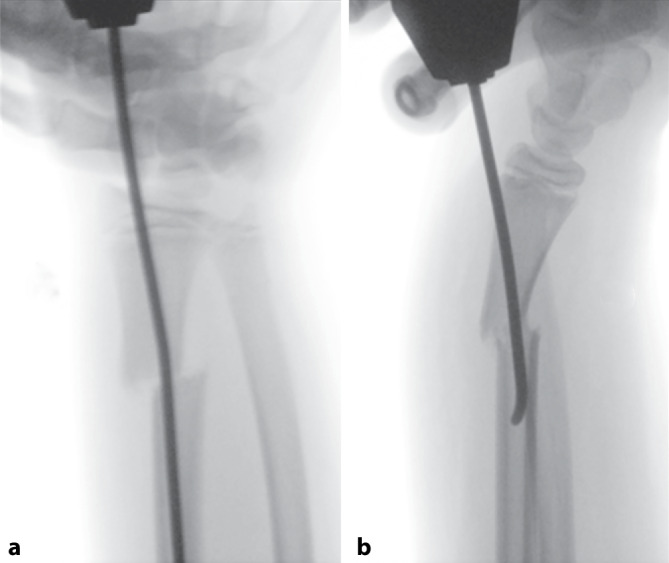
Instabilität der Versorgung mittels streckseitigem TEN (**a**). Palmare Abkippung des distalen Fragmentes durch streckseitigen TEN (**b**)

Die streckseitige Versorgung vom Tuberculum Listeri aus birgt das Risiko der Verletzung der Extensor-pollicis-longus(EPL)-Sehne [8] sowie der Dislokation des distalen Fragmentes nach palmar (Abb. 2).

Die optimale Versorgung dieser Region ist somit weiterhin nicht geklärt.

Eine alternative Technik stellt aus Sicht der Autoren die antegrade intramedulläre Nagelosteosynthese des Radius dar. Nach anatomischer Überprüfung der Technik folgte eine Nachuntersuchung an 30 Kindern [9]. Diese Arbeit orientierte sich an der initialen Technik mit Kirschner-Drähten von Sato et al. [10] und stellte eine Weiterentwicklung der Technik dar. Andere zeigten ein geringes Risiko- und Komplikationsprofil [11, 12].

Eine weitere Therapiealternative kann die S‑Technik mit einem retrograden TEN sein, wobei durch entsprechendes Vorbiegen eine Abstützung des kurzen distalen Fragmentes gelingen kann [13].

## Operationsprinzip und -ziel

Ziel der antegraden intramedullären Nagelosteosynthese ist die geschlossene Reposition und Retention in einer im Rahmen der Korrekturgrenzen befindlichen Stellung. Hierbei kommt es nicht zu einer 3‑Punkt-Abstützung, sondern zu einer antegraden Schienung des distalen Fragmentes, vergleichbar mit der retrograden Versorgung von subkapitalen Humerusfrakturen im Kindesalter mittels TEN [14]. Als Alternative kann auch ein vorgebogener Kirschner-Draht genutzt werden, der allerdings eine etwas geringere Stabilität bietet. Durch das Ausheilen in regelrechter Position werden die Voraussetzungen für die Wiedererlangung der Funktion und des kompletten Bewegungsumfang, insbesondere der Pro- und Supination, geschaffen.

## Vorteile

- Stabiles, minimal-invasives Verfahren zur Behandlung von diametaphysären Unterarmfrakturen
- Frühfunktionelle Nachbehandlung
- Schnellere Wiedererlangung der Sportfähigkeit im Vergleich zur konservativen Therapie
- Verhindern einer verfahrensassoziierten Dislokation (wie unter Vorbemerkungen erwähnt)

## Nachteile

- Relevantes Risiko der Verletzung des Ramus profundus und superficialis des N. radialis in der Hand des in dieser anatomischen Region Ungeübten
- Eröffnung der Kortikalis mit dem Pfriem kann erschwert sein, da der Knochen hier härter ist als in der Metaphyse
- Größere Schnittführung als bei den alternativen Verfahren notwendig

## Indikationen

- Frakturen, die nach Lieber et al. [5] definiert sind (Abb. 1)
- Stark dislozierte Frakturen, die konservativ nicht behandelt werden können

## Kontraindikationen

- Frakturen des distalen Unterarms
- Frakturen des Unterarmschaftes
- Komorbiditäten, die eine Operation verhindern
- Verletzungen der Haut oder kritische Weichteile im Zugangsbereich

## Patientenaufklärung

- Aufklärung von Kind und Eltern über Therapiealternativen (konservativ/ESIN retrograd/Kirschner-Drähte)
- Gegebenenfalls Notwendigkeit der offenen Reposition
- Allgemeine Operationsrisiken, wie z. B. Gefäß‑/Nervenschaden etc.
- Notwendigkeit der Metallentfernung
- Risiko der sekundären Dislokation
- Fehlstellung und Fehlfunktion
- Dauer der Konsolidierung
- Pseudarthrose
- Sportkarenz

## Operationsvorbereitungen

- Standardröntgen der Frakturregion in 2 Ebenen
- Klinische Evaluation der peripheren Durchblutung, Motorik und Sensorik (pDMS)
- Untersuchung der Weichteile
- Bildverstärker (BV)

## Instrumentarium

- TEN Stärke 2–3,0 mm
- Pfriem
- TEN-Applikator
- Parallelflachzange
- Schneideinstrument für TEN
- Gegebenenfalls 2,5-mm-Bohrer
- Gegebenenfalls Kirschner-Drähte 1,6/2,0 mm für Kapandji-Manöver

## Anästhesie und Lagerung

- Rückenlage mit ausgelagertem Arm auf Handtisch
- Freie Durchleuchtungsmöglichkeit mit Einblenden
- Desinfektion Hand/Unterarm bis handbreit proximal des Ellenbogengelenks
- Intubationsnarkose mit ggf. Relaxation bei grober Dislokation der Fraktur
- BV auf der Seite der Ulna bei proniertem Arm

## Operationstechnik

### Antegrade intramedulläre Nagelosteosynthese des Radius

**Abb. 3:**
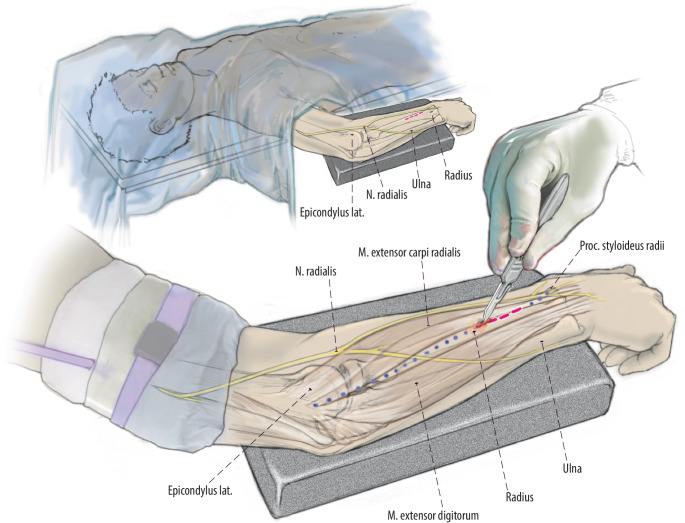
Zugangsweg eingezeichnet. Verlauf des Thompson-Zugangs *gepunktet* vom dorsalen Processus styloideus radii zum Epicondylus lateralis. Im mittleren Anteil dieser Linie lässt sich zwischen M. extensor carpi radialis brevis und M. extensor digitorum ein Softspot tasten. Hier erfolgt die Schnittführung über 2–3 cm. Ulnar vom Zugang verläuft der Ramus profundus und radial der Ramus superficialis des N. radialis in engem Bezug zur Muskulatur

**Abb. 4:**
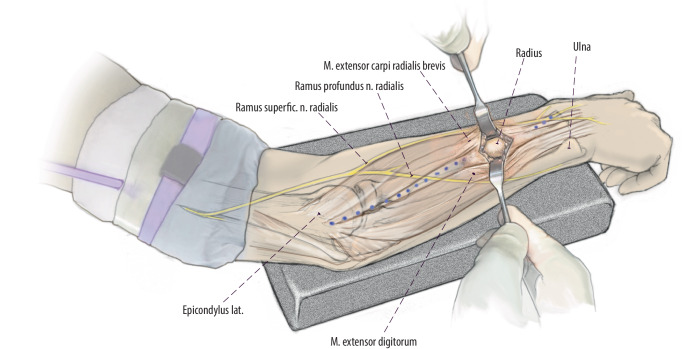
Schnitt eröffnet mit Langenbeck und Nerven. Schnittführung über 2–3 cm. Stumpfes Präparieren entlang der Muskellücke zwischen M. extensor carpi radialis brevis und M. extensor digitorum. Einsetzen von Langenbeck-Haken unter moderatem Zug zur Schonung der Nerven. Durch das stumpfe zur Seite halten der Muskelbäuche nach radial und ulnar und der in Bezug zu diesen liegenden Nerven kann eine Schädigung im Rahmen der Eröffnung der Kortikalis und der Insertion vermieden werden. Diese moderate Retraktion muss konsequent eingehalten werden, da das Risiko einer Verletzung des Ramus profundus und superficialis sonst deutlich erhöht ist. Darstellen der dorsalen Kortikalis

**Abb. 5:**
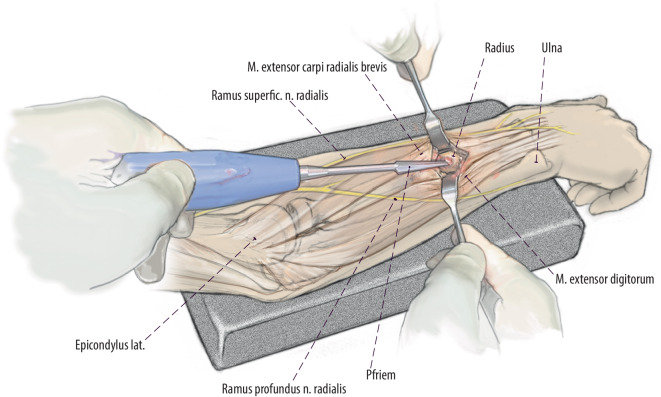
Perforation der Kortikalis unter Sicht. Bei mittels Haken offen gehaltener Wunde erfolgt die klassische Eröffnung der dorsalen Kortikalis mit dem Pfriem. Dies kann zeitweise sehr schwierig sein, da der Knochen hier härter als metaphysär ist. Wir empfehlen dann zunächst das Eröffnen der Kortikalis mit einem Bohrer der Stärke 2,5 mm mit Nutzung eines Gewebeschutzes. Im Anschluss dann weiteres Eröffnen, wie zuvor beschrieben, mit dem Pfriem ca. 45° zur Knochenoberfläche. Wichtig ist, wie zuvor beschrieben, die konsequente Schonung der Nerven durch Schutz mittels Langenbeck- oder Hohmann-Haken

**Abb. 6:**
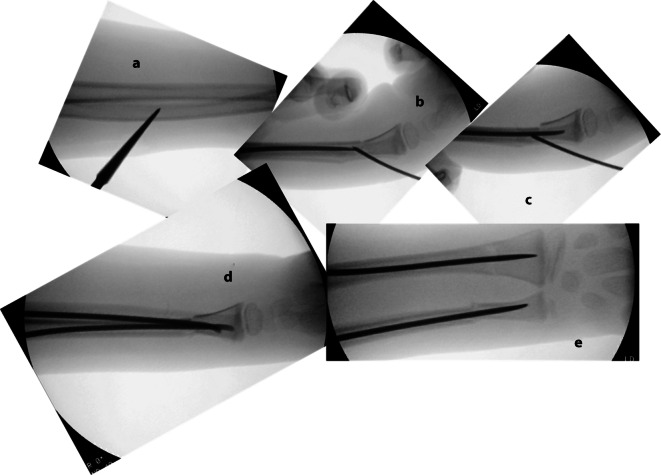
Intraoperative Bilder.** a** Radiologische Darstellung des Eingehens mit dem Pfriem im 45°-Winkel, da der Schaft hier einen kleinen Durchmesser hat, ist noch mehr darauf zu achten, nicht die Gegenkortikalis zu perforieren. **b** Vorschieben des TEN bis zur Fraktur unter drehenden Bewegungen, dann Reposition der Fraktur. Hier kann das in der Abb. 7 beschriebene Kapandji-Manöver hilfreich sein, um eine offene Reposition zu vermeiden. Ebenfalls sollte die Kufe des TEN mit der Flachzange begradigt werden. **c** Bei reponierter Fraktur Vorschieben des TEN bis knapp unter die Wachstumsfuge. **d**, **e** Additive antegrade Versorgung der Ulna und Ausrichten der TEN-Enden bis zur optimalen Frakturstellung

(Abb. 3, 4, 5, 6, 7 und 8)

**Abb. 7:**
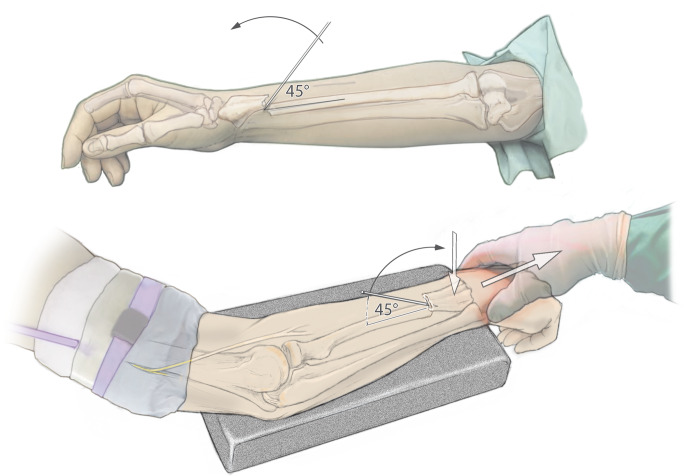
Kapandji-Manöver. Zur einfacheren Reposition kann von streckseitig ein Draht der Stärke 1,6 oder 2,0 mm im 45°-Winkel in den Frakturspalt eingebracht werden. Unter Längszug des Armes und Druck von dorsal auf das Fragment wird der Draht dann sukzessive nach distal gekippt, sodass es zu einer Reposition des Fragmentes kommt. Der Draht kann dann temporär in der Gegenkortikalis des Schaftes fixiert werden, bis der TEN nach distal vorgebracht wird. Anschließend wird der Draht wieder entfernt

**Abb. 8:**
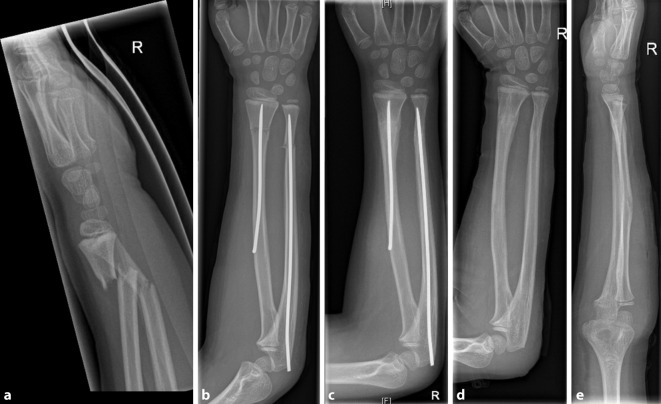
Fall eines 7‑jährigen Jungen nach Sturz vom Klettergerüst auf den rechten Arm und radiologische Darstellung einer diametaphysären Unterarmfraktur (**a**). Postoperatives Röntgenbild nach antegrader TEN-Osteosynthese (**b**). Regelrechte Konsolidierung nach 3 Monaten (**c**). Verheilte Fraktur in regelrechter Stellung nach Metallentfernung (**d**, **e**)

### Alternative Technik: S-Osteosynthese mit retrogradem TEN

**Abb. 9:**
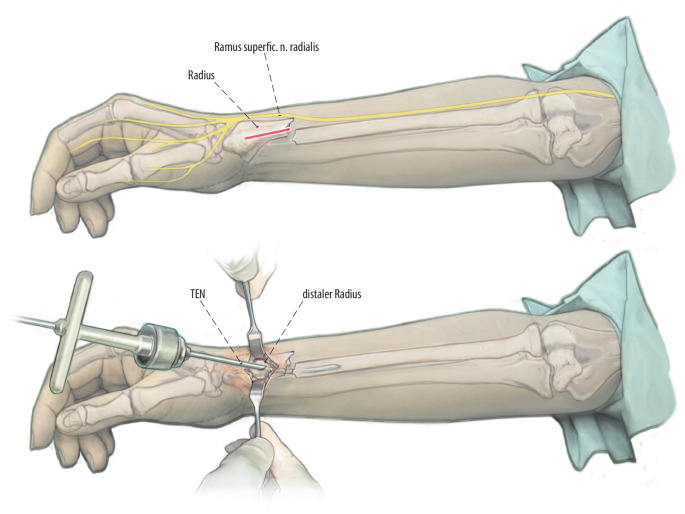
In Narkose wird als Zugangsweg der radiale Zugang am distalen Radius gewählt und in typischer Art und Weise das Einbringen des TEN vorbereitet. Hierbei muss auf einen ausreichenden Abstand zur Ranvier-Zone geachtet werden, was teils schwierig ist bei den kurzen Fragmenten. Dann wird der TEN zunächst unvorgebogen in den Markraum eingebracht und die Fraktur nach Reposition unter Bildverstärker überbrückt. Um die exakten Umkehrpunkte und eine optimale Reposition zu erreichen, wird der TEN zunächst bis zur optimalen Position nach proximal vorgetrieben, mit aktuell Inkaufnahme einer gewissen Dislokation im Frakturspalt

**Abb. 10:**
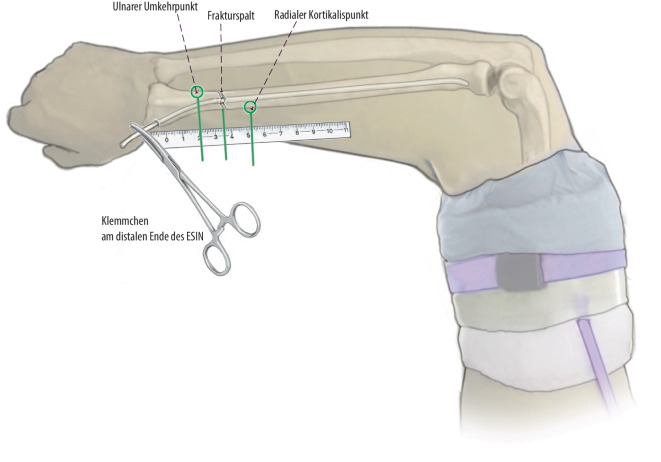
Anschließend wird ein Klemmchen am distalen Ende des ESIN befestigt, und unter Bildwandlerkontrolle werden exakt der radiale Kortikalispunkt proximal der Fraktur, der Frakturspalt und unmittelbar distal der ulnare Umkehrpunkt an der Kortikalis abgemessen

**Abb. 11:**
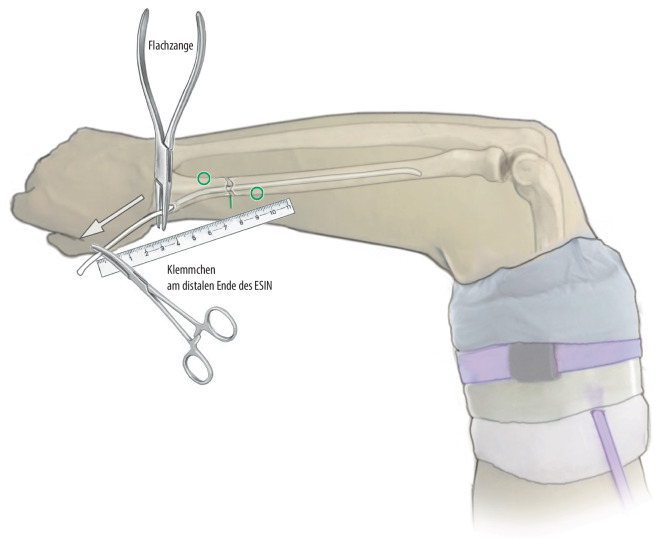
Anschließend wird der TEN etwas zurückgeschlagen, und in situ befindlich werden die erhobenen Punkte abgemessen und ein S eingeformt. Dabei ist die Richtung der Dislokation zu beachten

(Abb. 9, 10, 11, 12 und 13)

**Abb. 12:**
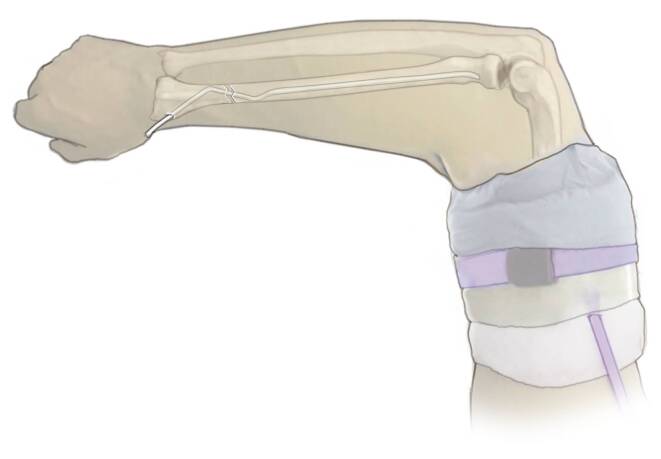
Der TEN wird dann wieder vorgeschoben In der Regel muss das S so gebogen werden, dass der proximale Umkehrpunkt sich an der radialen Kortikaliswand abstützt, die Schräge des S diagonal durch die Frakturlinie verläuft und der distale Umschlagpunkt sich sodann ulnar an der Kortikalis abstützt und anschließend nach radial lateral distal in dem Knochen verläuft. Hierdurch kommt es zu einer Restreposition

**Abb. 13:**
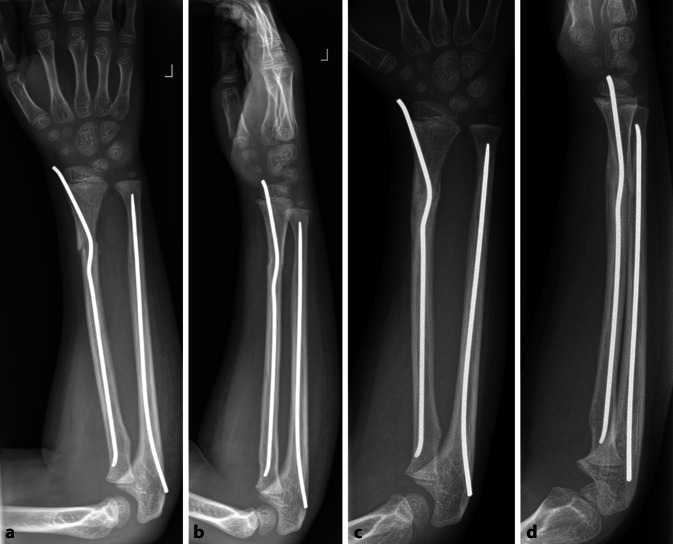
Fallbeispiel. Versorgung einer diametaphysären Unterarmfraktur mit einem doppelt gebogenen TEN radial und antegradem TEN in der Ulna direkt postoperativ (**a**, **b**) und nach Konsolidierung (**c**, **d**)

## Postoperative Behandlung

- Erhöhtes Lagern des Armes auf einem Kissen mit Kühlung
- In der Regel keine Immobilisation in Cast oder Schiene; zur Schmerzreduktion kann eine Unterarmschiene für 3 bis 5 Tage angelegt werden
- OP im Tagesverlauf ambulant möglich, ansonsten Entlassung am Folgetag
- Wundkontrollen und ggf. zeitgerechter Fadenzug, falls kein resorbierbares Material verwendet wurde (Ausnahme!!)
- Frühfunktionelle Nachbehandlung ohne Ruhigstellung
- Röntgenkontrollen direkt postoperativ oder nach 1 Woche sowie zur Konsolidierung nach 4 Wochen und vor Metallentfernung
- Sportkarenz für Vollkontakt für 8 Wochen („high impact“)
- Schwimmen, Joggen etc. nach 4 Wochen („low impact“)
- Materialentfernung nach Konsolidierung zwischen 3 und 6 Monaten

## Fehler, Gefahren, Komplikationen

- Bei fehlendem striktem Schutz der Nervenäste relevantes Risiko der Verletzung des Ramus profundus und superficialis des N. radialis
- Fehlende Möglichkeit der Reposition
  - Gegebenenfalls limitiert offene Reposition
  - Gegebenenfalls offene Reposition und Plattenosteosynthese als Reserveverfahren
- Postoperatives Hämatom → ggf. chirurgische Entlastung
- Irritation am subkutan liegenden Nagelende mit einer möglichen Serombildung
- Sekundäre Dislokation → Revisionsoperation mit ggf. Verfahrenswechsel
- Infektion → je nach Ausmaß und Dauer Notwendigkeit einer Wundrevision bis zu Implantatentfernung und Revisionsoperation im Verlauf
- Verzögerte Knochenheilung bzw. Pseudarthrose → Revisionsoperation

## Ergebnisse

Von 2012 bis 2022 wurden insgesamt 17 Kinder im Alter zwischen 5 und 16 Jahren in antegrader Technik operiert. Hiervon wurden 14 Patienten primär versorgt und 3 sekundär bei Fehlstellung mit additiver Korrekturosteotomie. Die Erhebung erfolgte retrospektiv. Nachbehandlungszeitraum mindestens 6 Monaten. Die Metallentfernung konnte in allen Fällen zwischen 3 und 6 Monaten erfolgen. Nach Ausheilung bzw. in der letzten klinischen Kontrolle zeigte sich bei allen Kindern ein volles Range-of-Motion im Seitenvergleich, insbesondere im Hinblick auf die Pro- und Supination. Klinisch wie auch radiologisch zeigten sich keine Fehlstellungen bei komplettem Remodelling der Fraktur. Ein Junge zeigte ein postoperatives Serom am TEN-Ende, welches bei der Metallentfernung entlastet wurde. Weitere Komplikationen, wie z. B. Nervenschäden, Wundheilungsstörungen oder Infekte, zeigten sich nicht. Ruhigstellungen erfolgten nicht. Der Return-to-Sports begann nach 8 Wochen.

In der alternativen S‑Technik wurden zwischen 2019 und 2021 5 Kinder operiert. Die Kinder waren zum Zeitpunkt der Operation 9,58 (± 2,92) Jahre alt. Alle Kinder wurden primär am Unfalltag mit dieser Technik versorgt. In keinem der Fälle wurde ein sekundärer Repositionsverlust beobachtet. Alle Frakturen heilten im Mittel innerhalb von 3 Monaten aus, und es erfolgte regelhaft die Metallentfernung. Hier lag keine Differenz zu anderen Frakturversorgungen in dieser Region vor. Die Metallentfernung gestaltete sich unkompliziert. Alle Kinder waren in der Folge beschwerdefrei und zeigten nach Entfernung des Materials ein volles Range-of-Motion im Ellenbogen und Handgelenk bei voller Belastbarkeit des Armes. Komplikationen traten nicht auf. Diese Ergebnisse und die Anwendbarkeit der alternativen Technik wurden auch bereits zuvor durch Krohn et al. [13] bestätigt.
